# Can an effective SARS-CoV-2 vaccine be developed for the older population?

**DOI:** 10.1186/s12979-020-00180-2

**Published:** 2020-04-11

**Authors:** Graham Pawelec, Nan-ping Weng

**Affiliations:** 1 Department of Immunology, University of Tübingen, Tübingen, Germany; 2 Health Sciences North Research Institute, Sudbury, Ontario Canada; 3 National Institute on Aging, Baltimore, MD USA

**Keywords:** Severe acute respiratory syndrome, SARS-CoV-2, Immunosenescence, Vaccination, COVID

## Abstract

The emergence of SARS-CoV-2 and its inordinately rapid spread is posing severe challenges to the wellbeing of millions of people worldwide, health care systems and the global economy. While many younger people experience no or mild symptoms on infection, older adults are highly susceptible to life-threatening respiratory and systemic conditions which demand a full understanding and leveraging of knowledge of the differences between immunity in young and old people. Consequently, we welcome papers addressing any issues relevant to immunity and ageing in the context of SARS-CoV-2, and will endeavour to fast-track peer-review. We aim to provide a platform exclusively for discussions of individual and age differences in susceptibility and immune responses to COVID caused by SARS-CoV-2 infection and how to prevent or reduce severity of disease in older adults.

## Introduction

The newly-emerged Coronavirus SARS-CoV-2 is highly infectious and has rapidly spread throughout the world with a case fatality rate (CFR) highly dependent on age and pre-existing conditions associated with age. As more data become available, average CFRs in children appear negligible, in young adults perhaps up to 1%, but in people over 60 rising to 4%, over 70 up to 9% and over 80 even up to 18% (see, for example, https://www.cebm.net/covid-19/global-covid-19-case-fatality-rates/). Clearly, these figures can vary tremendously from country to country, and depend on the state of health of the patients and the state of the health care system, but it is likely that this sort of age stratification reflects the general characteristics of Coronavirus disease (COVID). Obviously, there is vigorous debate as to why this may be, but a common denominator is the waning of immunity with age. Although much effort is currently directed at repurposing licensed drugs as anti-virals, particularly those with anti-inflammatory effects [1] this is usually viewed as a stop-gap measure and hopes are pinned on developing effective vaccines. An unprecedented world-wide effort is channelling academic and industrial resources into the rapid production and testing of SARS-CoV-2 vaccines. Quite apart from the potential dangers of accelerated vaccine development [2], and remembering the additional danger of immune enhancement [3], even were this successful the question remains whether the most susceptible members of the population (say, those over 60) would benefit.

### COVID immunopathology and potential interventions

There is no pre-existing immunity to a virus not previously encountered except via cross-reactivity or shared viral antigen, as is sometimes the case for seasonal influenza strains. As SARS-CoV-2 is almost certainly completely novel for at least the vast majority of people, the virus enjoys unrestrained entry into host cells which then rely on intracellular (cell-intrinsic) anti-viral defence mechanisms [4]. If these fail, cell death releases damage-associated molecules (DAMPs) and viral particles triggering inflammatory reactions. Severe acute respiratory syndrome is caused by dysregulated over-exuberant inflammatory responses that can progress to a systemic sepsis-like “cytokine storm” [5], which together with effects of the virus also directly infecting other organs, not just the lung, can result in multiple organ failure. The aim of prophylactic vaccination of course is to induce sufficient neutralising antibody to prevent infection and sufficient numbers of virus-specific resident memory cytotoxic T cells in the lung to prevent viral replication. This requires the presence and efficient cooperation of antigen-presenting cells, T cells and B cells within a correctly functioning microenvironment (lymph node). When vaccination is unable to elicit qualitatively or quantitatively sufficient protective antibody, host cell infection will still take place, and may trigger sequelae described above. Given the rapidity and degree with which SARS-CoV-2 can cause immunopathology in the lung, vaccines would have to be highly efficient in generating neutralising antibody as well as protective cell mediated local immunity to prevent this sequence of events. Achieving such immune protection by a vaccine is quite feasible in the young, but it may prove to be challenging in old populations as evidenced by the low efficacy of seasonal influenza vaccine in such populations. Alternatively, adoptive immunotherapy with neutralising monoclonal antibody, as in cancer treatment, may be a possibility, and several companies are working on this. However, repeated i.v. infusion of sufficient antibody does not seem a priori an optimal approach. Clearly, traditional vaccination to stimulate the patient’s own response would be preferable, but how likely is it that that could be accomplished in older people?

### Immunosenescence and its underlying mechanisms

Altered immune competence with increasing age, so-called immunosenescence [6], is the result of changes at multiple levels of the immune system over time. It includes the altered balance of immune cell production in the bone marrow resulting in reduced lymphopoiesis and increased output of myeloid lineage cells which are also functionally compromised. Thymic involution substantially reduces the output of naïve T cells and the TCR repertoire contracts over time. Although loss of circulating naïve B cells is less profound than naïve T cells, reduced BCR repertoire diversity with age is also well recognized. Furthermore, aging is associated with the dysfunction of innate immune cells like neutrophils at sites of infection possibly due to the poorer capacity of the adaptive immune system to reign in over-exuberant inflammatory responses. The ability to generate adaptive immune responses is compromised by dysfunction of antigen-presenting cells and disorganised and fibrotic lymph node architecture. Collectively, these changes prevent appropriate control of the initial inflammatory response and decrease the generation of an efficient and robust adaptive immune response which requires the production of large number of functional effector T cells and B cells. For all these reasons, protective responses to infection or vaccination tend to be on average lower in many older adults than in the young, but there is enormous inter-individual variation in people owing to the individual variations of genetics and the history of environmental exposures. Hence, two crucial questions are raised by these considerations: 1) how can we measure immune and physiological status in an individual in a clinically meaningful manner and 2) how can we intervene at the crucial checkpoints thus identified in order to restore appropriate immune function? Identification of biomarkers of protective or detrimental responses to a SARS-CoV-2 infection or vaccine and determination of the kinetic pattern of these biomarkers during the course of infection or vaccine response are critical to address these questions.

### Biomarkers and vaccine requirement

There are few precedents to assist us here. Efforts to develop a prophylactic vaccine or SARS-CoV-1 were shelved when the infection faded by itself. There are surprisingly few data available concerning the status of immune responsiveness to truly novel antigens in humans. A study on Yellow Fever vaccination of the elderly pointed to dysfunctional antigen-presenting cells and a dearth of antigen-specific CD4+ Th helper cells as culprits in the low antibody responses of older vaccinees [7]. Otherwise, we have to rely mostly on the large literature on seasonal influenza vaccination. However, the problem here is that everyone has already been exposed to some strains of influenza and even newly-emerging strains such as the avian H7N1 are not entirely novel. Nonetheless, knowledge garnered on immunity and responses to vaccination against this virus may tell us something about the capacity of older adults to respond to SARS-CoV-2. Primate models featuring the responses of older monkeys to SARS-CoV-1 infection may also be informative [8]. A systems biology approach will be needed to identify the protective antigen/epitopes of SARS-CoV-2 for both antibody/B cell responses and T cell responses and to classify them as protective or non-protective responses to serve as useful biomarkers. Importantly, vaccine design for the older adult should aim to stimulate a broad T and B cell response potentially overcoming reduced immune function in the older population.

### How can current immunological knowledge be leveraged to protect the oldest old against COVID?

According to the above arguments, we consider it unlikely that a conventional vaccine based on young adult responses will be highly effective in COVID prophylaxis for older adults, but should be rigorously applied to everyone else to achieve herd immunity that will indirectly protect the elderly. The ability to prevent infection by adoptive immunotherapy remains a possibility, albeit logistically and financially challenging. Pharmacological prevention of infection by other means, for example, by blocking the interactions between viral proteins and host cell molecules acting as receptors may be useful. Finally, various ways to improve the general immune functions in the older population should be considered and developed to strengthen the immune response to infection and vaccine in general. These approaches could include interventions at the level of hematopoiesis to correct the skewing of output towards dysfunctional myeloid cells responsible for acute inflammatory responses in the lung, normalisation of T cell progenitor output and reconstitution of the thymus for correct selection of T cells, especially regulatory T cells to keep inflammation in check, reconstitution of antigen presentation function in the lymph nodes and re-alignment of T-B cell interactions and functionality. In the meantime, the major benefit of vaccination will be seen at the population level in younger people. Once herd immunity is established, the well-known effect of diluting out new hosts for acute viruses should result in the virus disappearing, with the proviso that protective immunity is retained for long enough (this is not yet established) and reinfection is not introduced from a location where new hosts were still available. And with the linked proviso that the virus does not mutate into a form against which immune memory is not present.

We welcome papers addressing any of the issues discussed above and will endeavour to fast-track peer-review to provide a platform exclusively for discussions of individual and age differences in immune responses SARS-CoV-2 and susceptibility to COVID and how to prevent or reduce severity of disease in older adults.

## Data Availability

Not applicable.
